# 

**Published:** 1895-05-25

**Authors:** 


CONTENTS.

**Mr. Balfour on Medicine** - 123
**Drugs and Disease** - 124
**On Modern Progress in Ophthalmic Medicine and Surgery.** By R. BRUDENELL CARTER, F.R.C.S. - 125
**Modern Sociology.**—The Parents of Prisoners - 126
**Medical Progress and Hospital Clinics—**
Cataract as it is Met with in Ordinary Practice - 129
Certain Cases of Painful Menstruation - 129
PROGRESS IN MEDICINE.—Infective Diseases - 131
PROGRESS IN SURGERY.—Surgery of the Vermiform Appendix - 133
Surgery of the Liver and Gall Bladder - 134
**Annotations.**—A Doctor and his Fees—Opium-fed Infants—London Underground - 127
**Notes and News** - 128
**The Institutional Workshop:—**
THE FUNCTION OF A FEVER HOSPITAL - 135
PRACTICAL DEPARTMENTS. — The Blackman Rapid Drying System - 136
HOSPITAL FESTIVALS AND MEETINGS - 136
PRACTICAL POINTS - 138
**Editor's Letter Box** - 138
**The Book World of Medicine and Science** - 139

EXTRA SUPPLEMENT.—THE NURSING MIRROR.

**News from the Nursing World.**—The Prince of Wales—"Work Is as It Is Done"—The Midwives Bill—Women Inspectors—Opinions Differ!—Card Tricks up to Date—Long Hours—Ambulance Work at Shrewsbury—Pleading for the Children—Aberdeen District Nurses—Asylum Attendants—Training Essential at Lucknow—Short Items - liii

**Elementary Anatomy and Surgery for Nurses.** By W. MCADAM ECCLES, M.B., M.S., F.R.C.S. - lv

**Queen Victoria's Jubilee Institute** - lvi

**Appointments** - lvi

**Care of the Sick in Alexandria and Cairo** - lvi

**Novelties for Nurses** - lvi

**Everybody's Opinion** - lvii

**Indian Nursing Service Applications** - lvii

**Notes and Queries** - lvii

**Where to Go** - lvii

**The Book World for Women and Nurses** - lviii

**For Reading to the Sick** - lviii

